# The Epidemiological Impact of STIs among General and Vulnerable Populations of the Amazon Region of Brazil: 30 years of Surveillance

**DOI:** 10.3390/v13050855

**Published:** 2021-05-07

**Authors:** Luiz Fernando Almeida Machado, Ricardo Roberto de Souza Fonseca, Maria Alice Freitas Queiroz, Aldemir Branco Oliveira-Filho, Izaura Maria Vieira Cayres-Vallinoto, Antonio Carlos Rosário Vallinoto, Marluísa de Oliveira Guimarães Ishak, Ricardo Ishak

**Affiliations:** 1 Laboratório de Virologia, Instituto de Ciências Biológica, Universidade Federal do Pará, Belém 66.075-110, Brazil; ricardofonseca285@gmail.com (R.R.d.S.F.); alicefgarcia@gmail.com (M.A.F.Q.); ivallinoto@ufpa.br (I.M.V.C.-V.); vallinoto@ufpa.br (A.C.R.V.); marluisa.malu@gmail.com (M.d.O.G.I.); rishak@ufpa.br (R.I.); 2 Grupo de Estudo e Pesquisa em Populações Vulneráveis, Instituto de Estudos Costeiros, Universidade Federal do Pará, Bragança 68.600-000, Brazil; olivfilho@ufpa.br

**Keywords:** STI, HIV-1, HTLV, HBV, HPV, *T. pallidum*, *C. trachomatis*, epidemiology, Amazon region

## Abstract

Sexually transmitted infections (STIs) represent a worldwide public health problem and, although many of them are curable, they continue to be neglected, especially in areas with a low human development index, such as in the northern region of Brazil. This review describes the results of 30 years of studies at the Virus Laboratory at the Federal University of Pará, including the prevalence and molecular epidemiology of HIV-1, HTLV-1/2, HPV, HBV, *Treponema pallidum* and *Chlamydia trachomatis* among urban and non-urban populations, and also in vulnerable groups in the Brazilian Amazon. Control strategies and challenges in preventing STIs are discussed considering this immense geographic region, where essential health services are unable to reach the entire population, especially the most vulnerable, such as female sex workers, people who use illicit drugs, remnants of quilombolos and indigenous communities.

## 1. Introduction

**Figure 1:**
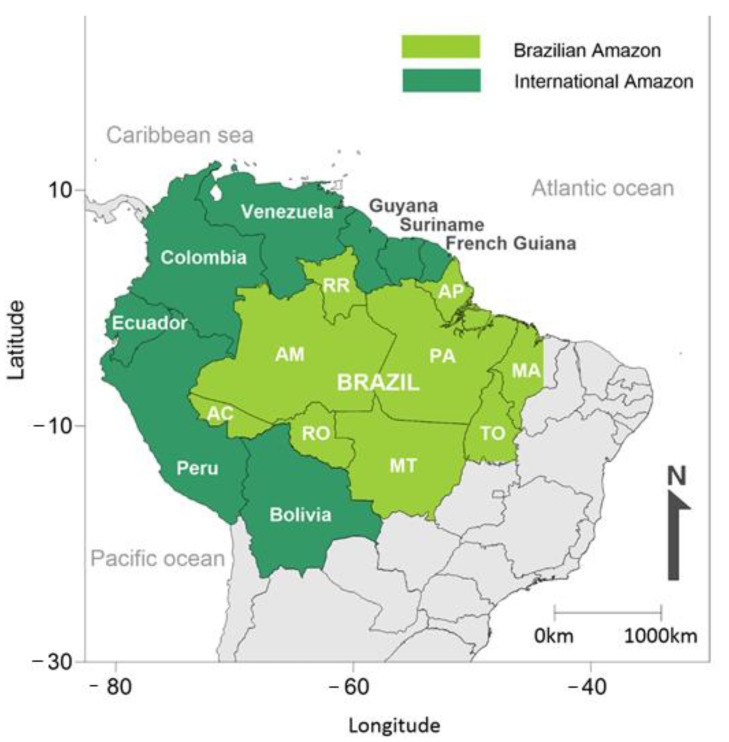
The Amazon rainforest extends over nine countries in South America (Brazil, Bolivia, Peru, Ecuador, Colombia, Venezuela, Guyana, French Guiana and Suriname). Around 60% of this tropical area is referred to as the Brazilian Amazon. Nine Brazilian states are part of this tropical region: Acre (AC), Amapá (AP), Amazonas (AM), Mato Grosso (MT), Maranhão (MA), Pará (PA), Rondônia (RO), Roraima (RR) and Tocantins (TO).

Sexually transmitted infections (STIs) represent a severe public health problem, especially among the poorest regions of the world, and cause a profound impact on reproductive and sexual health. The Amazon region stands out, spanning nine countries in South America (Brazil, Bolivia, Peru, Ecuador, Colombia, Venezuela, Guyana, French Guiana and Suriname). Around 60% of this tropical area is the Brazilian Amazon (Figure 1). Nine Brazilian states are part of this immense tropical region: Acre (AC), Amapá (AP), Amazonas (AM), Mato Grosso (MT), Maranhão (MA), Pará (PA), Rondônia (RO), Roraima (RR) and Tocantins (TO) [1]. These states present a human development index (HDI) below the national average, a vast territory [1] and poor access to health services and the control of diseases, especially sexually transmitted infectious agents.

According to the WHO, more than 1 million STIs are acquired daily worldwide. It is estimated that 38 million people are living with human immunodeficiency virus 1 (HIV-1) [2] and 5 to 10 million people are infected with human T-lymphotropic virus 1 (HTLV-1) [3]. About 300 million women carry human papillomavirus (HPV), the leading cause of cervical cancer and approximately 240 million live with chronic hepatitis B worldwide [4]. Each year, it is estimated that 6.3 million people are infected with *Treponema pallidum* and 127 million with *Chlamydia trachomatis* [2,5]. The spread of STIs is also amplified by parenteral and mother-to-child transmission during pregnancy, childbirth or the perinatal period, including HIV-1, HTLV-1/2, HPV, hepatitis B virus (HBV), *C. trachomatis* and *T. pallidum* [6,7]. The implementation of STI prevention and control strategies cost around USD 18.1 billion from 2016–2021 in 117 low- and middle-income countries, including Brazil [8].

This review presents the most relevant results of the impact of the infectious agents listed above obtained during the STI epidemiological and clinical research program developed at the Virus Laboratory (VL) of the Biological Sciences Institute of the Federal University of Pará in the last 30 years, with a focus in the Brazilian Amazon region. In addition to generating information about the epidemiology of STIs in the Amazon, we faced the challenge of controlling and preventing these infections in the largest geographic region of Brazil, but with the lowest human development indexes in the country.

## 2. Material and Methods

The first investigations of retroviruses (HIV-1 and HTLV-1/2) included seroprevalence information and looking for specific antibodies, where a screening test was used, usually ELISA tests and a confirmatory test, including Western blotting or indirect immunofluorescence. Subsequently, molecular biology methods such as nested PCR, restriction fragment length polymorphism (RFLP) and nucleotide sequencing were introduced to identify types and subtypes and for phylogenetic analyses.

For the study of HBV infection, conventional serological methods to detect HBsAg, anti-HBc (IgM and total antibodies) and anti-HBs markers were used as the common methodologies for detecting antigens and antibodies. HPV investigation included dot-blot hybridization, polymerase chain reaction, real-time polymerase chain reaction and nucleotide sequencing.

For the investigation of *T. pallidum* and syphilis, non-treponemal tests (VDRL and RPR) were used as screening methods, and treponemal tests (FTA-abs and ELISA) as a confirmatory method.

The isolation of *C. trachomatis* using cell cultures was restricted to the investigations during the 1980s and 1990s [9]. Since then, the confirmation infection has been based on the detection of short- and long-term antibodies (IgM and IgG) for the description of recent and past infections by performing indirect immunofluorescence using *C. trachomatis* serotype L2 as a substrate [9]. The discrimination of seroreactivity to *C. trachomatis* serotypes and discrimination with *C. pneumoniae* was by microimmunofluorescence [10]. Subsequently, immunoenzymatic assays [11] were made available for the detection of antibodies. In the 2000s, methods of detecting antigens in situ by immunohistochemistry [12] and nucleic acids were used to confirm infection with *C. trachomatis* [13,14].

The specific technical details for each infectious agent are described in the original publications that accompany the results and discussion of the review.

## 3. The Impact of HIV-1 in the Brazilian Amazon: From Description to Current Times

HIV-1 infection is a major cause of morbidity and mortality in the world and affects a portion of the adult population at the peak of their professional life, causing a great economic impact in many countries [15,16]. The prevalence of HIV-1 infection is higher in population groups that share common risk factors, including men who have sex with men, intravenous drug users, people in prisons and other closed environments, sex workers and trans people [17,18,19]. Transmission through sexual intercourse remains the most common, although there are several forms of prevention, including the use of condoms and prophylactic pre-exposure schemes (PrEP) [20].

By the end of 2019, over 38 million people living with HIV-1/AIDS (PLHA) were identified worldwide, with almost 1.7 million new infections registered at the time [21]. In Brazil, from 2007 to June 2020, there were 342,459 cases of HIV-1 infection, with 32,263 cases in the Amazon region [22]. The northern region of Brazil has 11.8% of the cases and an AIDS incidence rate of 24.4/100,000 persons, with the largest number of cases (43.47%) occurring in the state of Pará [22]. Four groups have already been identified (M, N, O and P), nine subtypes (A, B, C, D, F, G, H, J and K), a circulating recombinant form (CRF) and unique recombinant forms (URFs) [23]. In Brazil, subtypes B and F predominate, followed by subtype C and recombinant BF [24,25], except in the southern region, where subtype C is more common than subtype F [26]. In the northern region, subtype B is the most prevalent, and the state of Pará is an important gateway for entry of the virus into the region [27].

The first AIDS cases in Brazil occurred in the middle of 1982, in the city of São Paulo, in the southeastern region of the country, and with the description of HIV-1, the first questions were about the origin of the virus and if some populations described as having greater involvement in the epidemic could already have the infection. In the 1980s, the Virus Laboratory started seroprevalence studies of anti-HIV-1 antibodies, to verify whether the virus was already circulating in several urban population groups (general population, blood donors, male homosexuals and prostitutes) and indigenous people from the Xicrin tribe, among samples collected in the period between 1974 and 1980. The rationale was to investigate groups vulnerable to the acquisition of other STIs, that could already be hosting the virus, and indigenous communities that could provide answers about the ancestry of the infection. There was no evidence of HIV-1 infection, showing that spread of the virus probably did not precede the first cases recorded in Brazil [28]. Clinical and epidemiological information on viral infection were generated by other laboratories [29] located in the Brazilian Amazon and it became evident that the epicenter of the epidemic had been the southeast region and the virus rapidly spread to all other regions, including the Amazon region [30].

At the beginning of the epidemic, serological tests (known today as 1st generation) did not yet have great sensitivity and specificity, and the VL was involved in the evaluation of an agglutination test for the detection of anti-HIV antibodies, which could be used for screening in blood banks [31]. Although the prevalence of HIV-1 infection is well known in many locations in Brazil, most studies refer to large urban centers, especially those located in the south and southeast regions. Although HIV-1 has been isolated for more than 30 years, its occurrence in many municipalities located in remote areas of the Amazon region of Brazil is still unknown. The VL was the first in the Brazilian Amazon to describe the seroprevalence of anti-HIV-1 antibodies (0.6%) in the Tiriyo indigenous community (Karib linguistic group), which is located in the state of Pará, on the border between Brazil and Suriname, in the 1990s [32]. The low prevalence of HIV-1 infection in several indigenous communities in the Brazilian Amazon was also observed by other Brazilian groups [33,34].

The VL conducted the largest prevalence study of HIV-1 among the general population of four municipalities in the Marajó Archipelago (the largest river archipelago in the world), located in the interior of the state of Pará, showing low prevalence of anti-HIV-1 antibodies (0.64%) and confirming a greater circulation of the virus in the state capital [35]. This was corroborated recently, when a low prevalence of HIV-1 infection was also found in a region of the interior of the state of Pará with intense migratory flow due to the construction of a hydroelectric plant [36].

In addition to the general population, highly vulnerable groups were investigated for the acquisition of infection with HIV-1 and other STIs in the Brazilian Amazon region and in other states of Brazil. The VL conducted a pioneering study in the northern region of Brazil in the population of pregnant adolescents in the city of Belém, Pará and identified a low prevalence of HIV-1 infection (0.3%) [37], similar to that observed in the state of Amazonas [38]. The population of female sex workers (FSWs) is a highly vulnerable group for the acquisition of several STIs, and there are few studies on the epidemiology of HIV-1 in the Brazilian Amazon. A sharp increase in the prevalence of HIV-1 infection was observed from 2005–2006, from 2.3% [39] to 15.33% in 2017 [40], showing the spread of the virus towards the interior of the state of Pará over the years. 

Another vulnerable population for HIV-1 infection and of which little is known about the epidemiology of STIs are the people who use illicit drugs (PWUD) in the Amazon region. Recently, a high prevalence of HIV-1 infection (15.2%) has been identified in PWUD in the states of Pará and Amapá. The factors associated with the infection were lower educational level, lower income, crack cocaine use, a longer drug use history and a history of drug injection and engagement in unsafe sex, sex workers and large number of sexual partners [41]. In a collaborative investigation outside the Amazon, there was high prevalence of HIV-1 infection in elderly persons in the neighboring state of Piauí, showing that this group is also susceptible to HIV-1 infection and awareness of risky behaviors should be increased [42].

During the 1990s, the laboratory began investigating the co-infection of HTLV-1/HTLV-2 among individuals with HIV-1 in the city of Belém, Pará, as well as the impact on the clinical course of the disease. The first study revealed the occurrence of HIV-1/HTLV co-infection in 8%, with the prevalence of HTLV-2 being higher than that of HTLV-1 [43]. The population groups with major involvement were those who had the highest risk of sexual transmission, a different risk behavior from that seen in Salvador, Bahia, where the highest frequency of co-infection was with HTLV-1 and the main risk was associated with injected drug use [44].

HIV-1/HTLV co-infection studies aided by molecular biology tools, showed a decrease in the occurrence of co-infections over the years in the state of Pará, and the change in the profile of the predominant HTLV subtype. By 2005, the prevalence of co-infection was 3.5%, with a higher occurrence of HTLV-2c subtype [45] and in 2020, the prevalence dropped to 1.4% with the predominance of the HTLV-1a Cosmopolitan Group, Transcontinental subgroup [46]. Collaborative studies in the state of Piauí, Northeast Brazil, showed the occurrence of 1.61% co-infection, with HTLV-1a Cosmopolitan Group, Transcontinental subgroup being the most prevalent [47], showing the large regional differences in relation to the epidemiology of this co-infection.

Co-infections were continuously investigated by the VL in other geographical areas of the Amazon, including other STIs. A large variability of *C. albicans* morphotypes in the oral mucosa of HIV-1 carriers was identified, some of them were highly pathogenic [48] and subgingival sites, especially periodontal pockets, could act as reservoirs for Epstein–Barr virus (EBV) among HIV-1 carriers [49]. A low prevalence of cytomegalovirus (CMV) was observed in the oral mucosa of HIV-1 carriers with gingivitis and chronic periodontitis in the city of Belém, Pará [50]. A high prevalence of HBV co-infection among HIV-1 carriers was observed in the state of Piauí, with the identification of genotypes A, F and D [51] and a low prevalence of hepatitis C virus (HCV) in Belém, Pará [52]. HPV was detected in small proportion (26.8%), with the predominance of types of low oncogenic risk and a positive correlation with the decrease in the amount of CD4^+^ T lymphocytes in HIV-1 patients in Belém, Pará [53], but anti-*C. trachomatis* and anti-*C. pneumoniae* antibodies [54] and syphilis [55] were detected at high levels of infection. The investigations presented demonstrate the spread of other STIs among a population group who should be more cautious and oriented to avoid infections which can further compromise their immunological response and boost the inflammatory damage.

Among other groups of co-infections with HIV-1, it was shown that JC virus apparently has no impact on the number of CD4^+^ T lymphocytes and HIV-1 plasma viral load [56], while GB virus C (GBV-C, currently classified as pegivirus, HPgV1) was associated with a decrease in HIV-1 viral load, an increase in CD4^+^ T lymphocytes and a possible protective effect in the progression of infection to AIDS [57].

After describing key knowledge in the descriptive epidemiology of HIV-1 among population groups in the Amazon region of Brazil, the VL went on to describe the molecular epidemiology of HIV-1, in view of the existent gap at the time. It was shown that Belém, Pará, was one of the most important entry points for HIV-1 into the northern region, due to the great genetic diversity found in the city, between the years 1998 and 2002 [27]. Subtype B was already predominant, followed by subtypes F1, C, D and CRF02_AG, which was the first recombinant subtype identified in this region. During the same period, in the city of Macapá (Amapá), subtypes B and F1 were identified. However, some strains were characterized as B^env^/F^pro^, F^env^/B^pro^ and C^env^/B^pro^ mosaics [27].

The predominance of HIV-1 subtype B, as in most states of the Brazilian Amazon [58,59,60,61], was also observed in several population groups investigated by the VL. Subtypes B, F1 and C were among HIV-1-positive pregnant women under ART living in the state of Pará [62] and subtypes B and F1 and the BF1 mosaic among HIV-1 patients undergoing antiretroviral therapy [63,64]. Taken together, the identification of the diversity of HIV-1 subtypes suggests a low prevalence of resistance mutations to protease inhibitors, reverse transcriptase and integrase in HIV-1 strains circulating in the northern region of Brazil [62,63,64,65].

Progression to AIDS is associated with several factors related to the complex interaction between HIV-1 and the host. The VL started a unique line of investigation looking for gene polymorphisms of the immune and inflammatory responses which could be biomarkers influencing the progression of HIV-1 infection, including mannose-binding lectin (*MBL*), *IL6*, *IFNγ*, *IL-8*, *IL-10*, *TGFβ* and *FAS/FASL* genes. The first results obtained in relation to MBL (a serum protein important in the activation of the complement system) showed the presence of MBL*A, MBL*B and MBL*D alleles among HIV-1-positive individuals, with the MBL*B variant being associated with higher HIV-1 plasma viral load. This could lead to faster progression to AIDS, considering that a lower concentration of MBL protein in the serum can reduce the activation of the complement system and favor an increase in the plasma viral load of HIV-1 [66]. The investigation of the impact of -550 (H/L) and -221 (X/Y) mutations located in the promoter region of the MBL gene on CD4^+^ T lymphocytes and HIV-1 plasma viral load suggested that haplotypes related to medium and low levels of MBL protein in serum might have an important influence on the progression of HIV-1 infection [67].

The only published information on the polymorphisms of chemokine receptors (CCR5-D32, CCR2-64I) and chemokine (SDF1-3A) in the population of Belém, Pará showed that the frequency of CCR5-D32 mutation is similar between individuals with and without HIV-1. However, CCR2-64I and SDF1-3A polymorphisms were more frequent in the group of HIV-1-negative individuals, indicating a possible protective effect from HIV-1 infection [68].

The frequency of polymorphisms related to proinflammatory and apoptotic genes, including IFNγ (+874T/A), TNF (308G/A), IL-6 (-174G/C), IL-8 (-251A/T), FAS (-670A/G), and FASL (-124A/G), among HIV-1-positive individuals presenting different clinical outcomes (elite controllers, slow long-term non-progressors and progressors) showed three genetic allelic variants of IL6-174G/C, FASL-124A/G and FAS-670A/G polymorphisms related to disease progression and immunological and virological markers in cohorts of HIV-1-positive persons [69]. Polymorphisms in the FAS and FASL genes suggested that the FAS-670 polymorphism might be associated with apoptosis of T CD4^+^ lymphocytes after HIV-1 infection [70].

In the continuing investigation for more accurate biomarkers between genetic polymorphisms and clinical markers of progression to AIDS (including T CD4^+^ lymphocyte count and HIV-1 plasma viral load), the VL included TNFα-308G/A, IFN+874A/T, IL-6-174C/G, IL-10-1082A/G and TGFβ-509C/T polymorphisms among HIV-1 carriers. The results suggested that the presence of the IFN+874A allele confers susceptibility to HIV-1 infection and a decrease in the number of CD4^+^ T lymphocytes and the association of genotype 509TT of TGFβ with an increase in HIV-1 plasma viral load [71]. Finally, the CYP2B6 G516T polymorphism seems to affect the response to efavirenz treatment by reducing the quantity of T CD4^+^ lymphocytes among persons with a high degree of miscegenation who use antiretroviral therapy [72]. The amount of information presented is leading the VL to focus on the more promising results and expanding the investigated groups.

Understanding the variables that control the progression of infection to AIDS has always been one of the key points of research worldwide. Why do some individuals move quickly to AIDS (fast progressors), while others are slow progressors and others do not progress (elite controllers or viremia controllers)? The population of the northern region of Brazil is mixed and this produces strong impact on the genetic background of individuals, and could influence the outcome of HIV-1 infection. The VL studied a cohort to evaluate the genetic profile (polymorphisms of CCR5Δ32 and SDF1–3’A) and the plasma levels of eight cytokines (IL-2, IL-4, IL-5, IL-9, IL-10, IL-13, IL-17 and IFN-γ) in three groups of HIV-1-positive individuals, including viremia controllers 1 and 2 (VC1 and VC2) without TARV and a non-controller (NC) group. The results showed that the mean values of CD4^+^ T lymphocytes were higher among VC than NC, but significantly lower than HIV-1-negative controls and the ratio CD4^+^ T lymphocytes/CD8^+^ T lymphocytes could be used as a biomarker for the VC groups. In addition, a clear signature indicated a change from Th1 to Th2 cytokine profiles between VC and NC groups [73]. The investigation of circulating virus showed higher selection pressure on mutations among the virus controllers and a higher frequency of immunological escape mutations in the genes investigated (*gag*, *nef*, *rev*, *tat*), with the description of two new mutations in *gag*. In conclusion, progression to AIDS is a result of the sum of characteristics and pressures exerted by the virus and the interactions with the host immunogenetic characteristics [74].

## 4. HTLV-1/2 in the Brazilian Amazon: From Human Migration to Associated Diseases

HTLV-1 and HTLV-2 are mammalian retroviruses with similarities in their biological and molecular properties [75]. HTLV-1 is associated with fatal diseases, including an aggressive neoplasm (adult T cell leukemia/lymphoma, ATL) and serious inflammatory diseases such as HTLV-1-associated myelopathy (HAM), uveitis, dermatitis and arthritis, among other conditions [76]. HTLV-2 is less strongly associated with diseases, but it has been used in the study of human migration markers [77]. HTLV-1/2 are associated with cells present in organic fluids such as blood, semen, vaginal fluid and breast milk and they are transmitted through sexual, parenteral and mother-to-child pathways (in utero, during childbirth and mainly breastfeeding) [78].

In the context of populations in the Amazon region, the VL defined the endemic transmission of HTLV-2 among indigenous peoples of the Brazilian Amazon [79]. Seroprevalence variations of anti-HTLV-1 and anti-HTLV-2 antibodies in 25 indigenous communities of different ethnicities and language groups showed the occurrence of a new molecular subtype (HTLV-2c), with sexual transmission of the virus as the main transmission route and intra-familial transmission of HTLV-2 in Kayapó villages, with clear evidence of mother-to-child transmission. This form of transmission was confirmed with the use of molecular biology methods in Kararô village (Kayapó), showing 99.9% genetic similarity in the sequence of the 5’LTR genomic region of the proviral DNA, between the mother and child infectious strains [80]. The work has been continued up to the present through periodic epidemiological surveillance showing the hyperendemicity of HTLV-2 in other villages of the Kayapó group such as Xikrin do Kateté, Djujeko, Oodjã, Aukre, Kendjan and Pukararankee [81,82]. In contrast, the high prevalence of infection has a very heterogeneous profile in indigenous populations as a result of the processes of the founding effect [83,84,85]. Epidemiological surveillance has been of great importance and the continued absence of infection in the villages of Araweté and Asurini after 36 years of follow-up is notable [86].

The introduction of molecular biology methods made it possible to perform the complete sequencing of the HTLV-2c genome, initially described among the Kayapó and causing hyperendemic infection in the Brazilian Amazon area [79], in order to look for genomic evidence that could explain the attenuation of the pathogenesis of HTLV-2 in relation to HTLV-1 [87]. *Tax* gene sequencing showed that HTLV-2c strains identified in urban and indigenous areas of the state of Pará presented the same mutation in the stop codon, identified as a unique aspect of HTLV-2c and which distinguishes it from the HTLV-2a subtype [81].

It was later shown that in addition to indigenous populations, HTLV-2c is already present in urban (blood donors and HIV-1 carriers) and rural populations in the states of Pará and Amazonas [43,82,83,88,89]. In Brazil, HTLV-2c was described in other areas, including São Paulo [90], Rio de Janeiro [91], Belo Horizonte [92], Porto Alegre [93] and Salvador [94]. It is worth mentioning that the virus has been described in Senegal, showing the great capacity of infectious agents to spread [95]. The presence of HTLV-2c in Senegal could be a result of the return of quilombos refugees that occurred soon after the abolition of slavery, to their motherland in Africa following intense interaction with local indigenous peoples [96]. In addition to HTLV-2c, the molecular subtype HTLV-2b was also described in the general population of the city of Belém [97] and among drug users in different municipalities in the state of Pará [98].

HTLV-1 infection in the Amazon has been mostly represented by the presence of the subtypes HTLV-1 transcontinental cosmopolitan and HTLV-1 Japanese cosmopolitan [99,100,101]. The introduction of HTLV-1 in the Amazon region probably occurred with the human migratory processes resulting from the slave trade from Africa between the 16th and 17th centuries and from the most recent Japanese migration to the Amazon in the late 18th and early 19th centuries [77,79]. The introduction of HTLV-1 from the slave trade is suggested by the presence of the virus in quilombola communities in the region [77,96]. Other routes of transmission, such as sharing of instruments for the use of injected drugs, have also been investigated and the prevalence was 3% for HTLV-1 and 2.3% for HTLV-2 (2.3%) in the state of Pará [98]. It is worth mentioning that the circulation of the molecular subtypes HTLV-1 transcontinental cosmopolitan, HTLV-1 Japanese cosmopolitan and HTLV-2c and the presence of HTLV-2b as a molecular variant were introduced by injected drug users.

The main dissemination of HTLV-1/2 through the sexual route characterizes it as one of the important STIs, of which carriers’ sexual behaviors act as facilitators for transmission, i.e., sexual contact without the use of condoms and multiple partners [102]. This was initially shown among HIV-1-positive individuals co-infected with HTLV-1/2, where the main risk factor reported was sex with multiple partners [43]. Among drug users, unprotected sex, the presence of ulcers and wounds in the genitalia and more than 12 sexual partners were factors associated with infection by both viruses [98].

The first cases of disease associated with HTLV-1 in the Amazon region were cases of HAM [103]. Since then, the VL has sought numerous markers of the immunological and inflammatory response [104,105] suggesting the association of genetic factors that lead to the intra-family distribution of the virus [106] and of the progression of disease in the development of HAM and other conditions of inflammatory disease among those infected with HTLV-1 [107,108]. Lung disease has also been reported in patients with HTLV-1 living in the state of Pará [109].

The large geographical distribution of HTLV-1 and HTLV-2 in the Amazon region poses a great challenge to describe the prevalence of HTLV-1/2 [85]. The geographical challenge associated with infrastructure limitations has made it difficult to determine the exact prevalence of infections, even though the virus has been described in several different populations of the Amazon region of Brazil for almost 30 years. As prophylaxis progresses towards a future HTLV-1 vaccine, it is important to determine who is at risk of being infected and possibly developing one of the diseases associated with HTLV-1 in order to implement preventive measures [85].

Prevalence rates and molecular epidemiology of HTLV-1/2 (and HIV-1) are shown in Table 1, which summarizes the results obtained in the different studies in the Amazon region of Brazil.

## 5. HPV: An Infection with a Chronic Disease Outcome in the Brazilian Amazon

Some HPV serotypes are sexually transmitted and some are associated with the etiology of cervical cancer, and other types of cancer, including cancer of the anus, vulva, vagina, penis and oropharynx [110]. More than 220 HPV genotypes have been identified [111] and classified as low and high oncogenic risk [112]. In Brazil, the virus is the etiology of approximately 5.1% of neoplasms in general, and almost 100% of cervical tumors, 88% of anal tumors and 50% of penile tumors. Regarding cervical cancer, there are an estimated 570,000 new cases per year [113].

Although it is one of the most common STIs worldwide, the epidemiology of HPV infection in the Amazon region of Brazil is still poorly known. Among indigenous populations of the Brazilian Amazon region, 22% of infections were observed among women of the Parakanã tribe [114] and 39.7% in indigenous communities in the state of Amazonas [115].

Most of the studies investigating the epidemiology of HPV in the northern region of Brazil were among uterine cervical samples from women with or without cervical cancer. In the city of Belém, Pará the prevalence of HPV among women with lesions of the uterine cervix showed a prevalence of 70.3% among women with invasive epidermoid carcinoma or adenocarcinoma, 63% in patients having either cervical intraepithelial neoplasia grade II or III and 36.8% in women with chronic cervicitis. In all groups, there was a predominance of subtypes 16 and 18, in addition to the presence of HPV types 31, 33, 45, 52, 58, 59 and 73 [116]. The ethnicity-related distribution of HPV-16 variants was investigated in biopsies from women with cervical intraepithelial neoplasia grade III or invasive cervical cancer. The most prevalent HPV-16 variant was the Asian-American B-2, followed by the European B-12 and the European prototype [117]. Among females living in riverside communities in the state of Pará, HPV prevalence was 16.4%, with 2.3% HPV-16 and 1.4% HPV-18 [118].

There is not sufficient epidemiological information on HPV among vulnerable FSWs. In the only study performed in the state of Amazonas [119] 100% HPV infection was observed among FSWs and, although the sample size was small, it is indicative of the possible impact of the infection.

Among the female prisoner population group in the municipality of Ananindeua, Pará, the prevalence of cervical HPV was 10.5% [120], while in riverside women in the state of Pará, the prevalence was 16.4%, with the identification of types 16 and 18 [121]. In women in rural and urban areas in the state of Pará, the prevalence of HPV infection was very similar, with 15% in urban areas and 14.2% in rural areas [122]. In the rural areas of the city of Coari, Amazonas, a prevalence of 19.1% of HPV DNA was identified in a cervical cancer screening study [123]. Female university students from Belém showed a 25.3% prevalence of HPV and the most prevalent genotype was HPV-61 (5.3%), followed by HPV-82, HPV-16, HPV-59 and HPV-6 [124]. Among women with autoimmune diseases, the prevalence was similar (22.8%), with the higher frequencies of genotypes HPV-58 (37.5%) and HPV-31 (31.3%) [125].

In the city of Belém, Pará, the oral cavity has been investigated and HPV was detected among 24.1% of individuals without clinically diagnosed lesions, infected with genotypes HPV-6, HPV-18 and HPV-58 [126]. The prevalence of HPV in the oral cavity of people who use crack cocaine was 39.9%, infected with genotypes 6, 11, 16, 18, 31, 33, 45, 52 and 58, with 12.6 % presenting multiple infections [127].

The presence of cervical and anal HPV infections is common and identification of cervical cancer might contribute to the prevention of anal cancer in women, especially in women living with HIV-1 [128]. The VL identified the types HPV-6, HPV-11, HPV-16, HPV-53, HPV-58, HPV-59, HPV-61, HPV-62, HPV-66, HPV-70, HPV-71 and HPV-102 as causing anal infection among women with HIV-1 in the city of Belém, Pará [53].

## 6. HBV: The Impact of the Geographical Area on the Prevalence of the Virus in the Brazilian Amazon

Hepatitis B virus (HBV) is associated with acute and chronic diseases, including cirrhosis, liver cancer and death [129]. According to the prevalence rates of the serological marker of viral persistence (HBsAg), the endemicity of the virus is classified as low (<2%), intermediate–low (2% to 4%), intermediate–high (5% to 7%) and high (8%) [129]. The Brazilian Amazon is characterized as a region of high endemicity [130,131], although it is not uniform. High frequencies of HBV-associated diseases and its sequelae have been recorded more frequently in the states of Acre, Amazonas and Rondônia than in other areas of the Amazon region [130,131]. A high lethality rate was associated with hepatitis delta virus (HDV) co-infection among HBsAg carriers in the state of Amazonas [130,132,133]. In Brazil, up to 2018, 233,027 cases of hepatitis B were reported, with approximately 14% of cases concentrated in the Amazon region [134]. HBV is prevented by safe and effective vaccines that prevent the development of chronic diseases and liver cancer [135,136].

The initial investigations of HBV by the VL were a consequence of the epidemiological information at the time about the extensive spread of the virus and its association with a new infectious agent described in Italy, HDV [133,137,138].

In the 1980s and 1990s, the VL conducted the first seroepidemiological investigation of more than 4000 people residing in seven urban communities in the state of Pará and 24 indigenous communities in the Brazilian Amazon, residing in six states of the region [139] (Table 2). Urban communities showed a clear-cut difference between seroprevalence rates, which were higher in the westernmost areas of the state of Pará (13.2–15.9%) than those located along the mouth of the Amazon river (6.7–11.8%) [139]. The prevalence of HBV markers among 2222 indigenous people ranged from 3.4–59.2%. In two communities investigated in the years 1983 and 1985, HBV infections were not found. Unfortunately, however, in 1990, the virus had already entered the community and established persistence in about 10% of the group [140].

For the first time, it was possible to show evidence that indigenous communities could modulate HBV infection differently than among urban population groups. The endemicity of the virus at high levels is usually accompanied by high levels of viral persistence. However, some groups were exposed in different ways, but there was no correlation with the levels of persistence. Differently from urban populations, the difference in modulation of HBV infection could be related to the low genetic variability of epidemiologically closed communities when compared with urban tri-hybrid population groups of the Amazon region [139]. The persistence of HBV in epidemiologically closed communities is worrying, since we are dealing with an agent that can be aggressive in terms of outcome (leading to cirrhosis and liver cancer) in a population of enormous vulnerability and without appropriate access to health services.

With the use of molecular biology methods, the investigation of HBV genotypes circulating among virus carriers treated at a university hospital was initiated and showed the predominance of genotype A (89.1%), followed by F (8.7%) and D (2.2%), although there was no influence of genotypes on the clinical manifestations or severity of liver disease [121]. The presence of pre-core and core mutations was detected at low levels and showed no association with the disease staging caused by HBV.

Epidemiological investigations among high-risk groups (FSWs and PWUD) showed prevalence rates of HBV infection ranging from 3.0% to 13.7% among FSWs that offered their services in municipalities and riverside communities in the state of Pará [121,141]. The rate of vaccine immunity (anti-HBs only) ranged from 0.7% to 4.7% [141,142] and genotype A (69.0%) was the predominant one [143]. Reduced monthly income, low education, unprotected sex, multiple sexual partners, unsafe sexual practices, illicit drug use and co-infection with other STIs have been associated with exposure to HBV [141,143]. These findings indicate the need for urgent measures to control the spread of HBV and other STIs and to promote the health of FSWs. The inclusion of these women in vaccination programs and other initiatives in the area of women’s health, including periodic gynecological examinations and testing for STIs, provision of condoms and other measures that promote self-care, will help to minimize the impacts on this group of vulnerable people, as well as in the general population.

Immunogenetic studies conducted among patients with chronic HBV residing in the state of Pará have been quite successful in showing associations between the polymorphisms investigated among the immunological system genes (*IFN-y*, *TGF-beta1*, *IL28b*, *MBL*, *PrtCR*, *IL-10*, *TLR-3*, *TLR-4* and *FoxP3*) and HBV infection. Higher serum levels of IFN-c and TGF-beta1 have been associated with chronic hepatitis B, and lower serum levels of IL-10 have been found in patients with active disease. The presence of the *TNF-a* 308 polymorphism allele A suggests a risk of progressive disease [144]. Single nucleotide polymorphisms (SNPs) in the *IL28b* gene indicated there is no association with the susceptibility and clinical evolution of hepatitis B [145]. SNPs in the *TLR3* and *TLR4* genes appear to be associated with higher levels of ALT, AST and prothrombin, in addition to being associated with higher levels of GGT [146]. 

In the investigation of the main SNP in the *MBL* gene, patients with active HBV who had wildtype AA genotype had a positive correlation between increased levels of transaminases, HBV DNA and the presence of mild to moderate fibrosis [147]. Mutations in the *FoxP3* gene, SNP-924 A > G, showed that G carrier status was associated with altered viral loads and liver enzyme levels in patients with chronic active hepatitis B (CHB-A) with inflammation and fibrosis. However, the frequencies of SNP-3279 C > A, A and-924 A > G, G were not directly associated with the histopathological profiles of the patients investigated [148]. It was also observed that the hepatic levels of mRNA expression of the *NGF* and *p75NTR* genes decreased and increased, respectively, in relation to the stage of inflammatory activity in HBV carriers. A positive correlation between the expression of the *p75NTR* and *NGF* genes was observed in livers with mild and moderate fibrosis, although not in cases of severe fibrosis and cirrhosis [149].

Providing efficient services and adequate resources for the diagnosis, treatment and prevention of HBV infection must be extended to all populations in the Brazilian Amazon. The concentration of health services and resources in the main urban cities of the states, the absence or irregular development of strategies to promote health in rural and remote areas (including riverside communities and indigenous tribes), risk behaviors and the low socioeconomic status of the population are relevant factors for the epidemiological scenario of HBV infection in the Brazilian Amazon. They are relevant variables, that if not dealt with, will keep HBV circulating at high or intermediate endemicity in the region over a long period of time.

## 7. The Not Yet Defined Impact of *Treponema pallidum* in the Brazilian Amazon

Syphilis is an STI caused by *T. pallidum*, an agent transmitted mainly by contact with infectious wounds on genital organs, in addition to blood or mother-to-child transmission during pregnancy, which includes the epidemiological clinical cases of acquired syphilis in pregnant women and congenital cases [150]. Although syphilis is a treatable and easily diagnosed disease, nearly one million pregnant women presented with syphilis in 2016, resulting in more than 200,000 newborn deaths [151]. In Brazil, in 2020, 152,915 cases of acquired syphilis were registered, with 61,127 cases of syphilis in pregnant women, 24,130 cases of congenital syphilis and 173 deaths from congenital syphilis [152].

The investigation of syphilis and *T. pallidum* by the VL started in the 1980s, through the screening of milk donor mothers in a public hospital milk bank (unpublished information). Subsequently, the first epidemiological investigation showed that in the general population of Belém, 15.8% was already infected with *T. pallidum*, and 4.4% had an ongoing infection [153]. The absence of the use of condoms during sexual intercourse and the lack of knowledge of syphilis as an STI were the most reported variables among those investigated.

With the approval of new projects that aimed to define the prevalence of STIs, two population groups of crucial importance as reservoirs of *T. pallidum* were our objects of investigation: HIV-1 carriers and FSWs, among whom an increase in the incidence of syphilis was demonstrated [154]. In a sample of 430 HIV-1 carriers, the prevalence of past infections by *T. pallidum* was 27.2%, and 7.7% had laboratory results compatible with ongoing syphilis [54]. The use of non-injectable illicit drugs, having more than one sexual partner per week, the practice of anal sex and homosexual/bisexual behavior were associated with exposure to *T. pallidum*. The greatest importance of this study was in the investigation of a population group that should already be looking for basic preventive measures to avoid the transmission of other STIs. In another study with HIV-1 carriers, who were ART naive, the prevalence of *T. pallidum* was 17.3% [55].

Among FSWs who lived in three urban locations in the northeast of the state of Pará, the prevalence of syphilis was 14.1%. This infection was associated with the offering of sexual services in low-income areas, a consequence mainly of the low level of education. The practice of anal sex in the first years of sexual intercourse was another important factor associated with syphilis. This proved the urgent need to establish more effective prevention and control actions for the population groups [155].

Recently, an epidemiological report with 180 FSWs offering sexual services in 25 locations in the Marajó Archipelago showed that syphilis was clinically present in 41.1% of the women investigated [156]. In addition to the low levels of income and education, the use of illicit drugs, a long period of prostitution and the lack of regular medical appointments were factors associated with exposure to *T. pallidum*. The molecular approach to detect the presence of point mutations (A2058G or A2059G) in the *T. pallidum* 23S rRNA gene showed that 23.5% of FSWs were infected with *T. pallidum* strains with point mutations indicative of resistance to treatment with macrolides. It is important to highlight the increase in infection levels among a population group with high vulnerability, living in places with a low human development index and lacking basic services and reaching women who act as reservoirs for the dissemination of the agent. The presence of *T. pallidum* among FSWs represents a point of great relevance for the extensive dissemination of the agent to FSWs and the avoidance of reaching a point of difficult control in the entire population of the archipelago.

The STI investigation approach among the general population of four municipalities in the Marajó Archipelago also included seroprevalence of *T. pallidum* and syphilis. It showed a level of 8.5% of previous exposure to the bacterium and 4.6% of recent infections [157]. The distribution of exposure to *T. pallidum* was found to be among young adults, gradually increasing with age by four times (2.2% to 9%). Young adults represent the main group of sexually active individuals and the presence of syphilis in this group is worrying, as it indicates a serious failure in the measures for the prevention and control of syphilis in the Marajó Archipelago.

## 8. Infection with *C. trachomatis* in the Brazilian Amazon and Associated Diseases

Human diseases associated with infections by bacteria of the genus *Chlamydia* are worrying public health problems. *C. trachomatis* is related to eye infections, such as trachoma and inclusion conjunctivitis, and genital infections, such as venereal lymphogranuloma, non-gonococcal urethritis, epididymitis, proctitis, salpingitis and mucopurulent cervicitis, which can progress to pelvic inflammatory disease, ectopic pregnancy and infertility [158,159,160,161]. The WHO estimates that there are more than 127 million new cases of *C. trachomatis* annually [2,5], which cause additional harm to thousands of women who become infertile [162,163,164]. The bacterium has well-defined forms of transmission, and the sexual route is probably the most common, making it one of the most important STIs.

Among the 19 serotypes of *C. trachomatis*, A–C are causes of eye infections, D–K cause urogenital infections and L1–L3 cause cases of venereal lymphogranuloma [165,166]. The prevalence of genital infections and their complications, by *C. trachomatis*, in developing countries is not clearly defined and the same is true in Brazil. The absence of laboratory diagnosis of the infection, the lack of notification of cases, the inappropriate use of antibiotics and the failure to apply preventive measures are important variables for the spread of the bacterium.

A study of *C. trachomatis* started at the VL in the 1980s confirmed the presence of antibodies among three specific population groups in the Brazilian Amazon [167]. Different prevalence rates were found in Belém (53.6%), gold mining areas (76.2%) and among the indigenous people of the Xicrin tribe (51.3%). The expansion of the investigated geographical area and the number of groups showed the spread of *C. trachomatis* among urban populations (prenatal clinics, gynecological clinics, STI clinics and FSWs) and indigenous populations (Parakanã and Kubenkokre) with prevalence ranging from 33.3% to 97.1% [9]. It is worth mentioning that the use of McCoy cell cultures for the isolation of the bacterium showed that *C. trachomatis* was the most important etiologic agent (30%) of non-gonococcal urethritis in an STI clinic and also infected asymptomatic women in gynecology and obstetrics clinics [9] in similar percentages previously found in other regions [168].

One of the focuses of the laboratory became the description of the involvement of the bacterium among isolated vulnerable indigenous human populations. A seroepidemiological study involving 2,086 people from 27 indigenous communities in six states in the Brazilian Amazon showed an average prevalence of antibodies of 48.6% and communities which never came into contact with the bacterium; 14 communities showed prevalence rates of up to 50%, six with up to 70% and nine that reached up to 90.7% [10]. It was also shown that 1.2% showed evidence of recent infection and 6.1% indicated they had persistent infection with *C. trachomatis*. Using microimmunofluorescence, seroreactivity showed infection by *C. trachomatis* serotypes associated with trachoma and STIs. Interestingly, some of the samples showed reactivity by indirect immunofluorescence, but they did not react with any of the antigens of *C. trachomatis* or *C. pneumoniae*, possible evidence that some community members were infected with other species of *Chlamydia* circulating in nature. Now, when emerging infections cross the inter-species barrier and are transmitted from animals to humans, such as HIV-1 [169], HTLV-3 and HTLV-4 [170] and SARS-CoV-2 [171], the information becomes even more up to date and relevant since we are dealing with vulnerable populations with little access to the health system.

Although there was no evidence of a geographical association with the distribution of prevalence, an interesting epidemiological pattern was present in that communities modulated prevalence levels differently from how *Chlamydia* was maintained in persistence as previously reported by the VL for HBV [139]. Communities could have low, medium or high levels of infection, and combine low, medium or high levels of persistence (Table 3), which indicated the influence of the genetic component of isolated and semi-isolated indigenous populations [10]. The high prevalence and high rate of persistence of *Chlamydia* in small indigenous communities are factors of great concern, since among closed communities, it is common to have a greater spread of STIs, including by other means of transmission than sexual routes [172,173]. It is worth mentioning that this is a worrying situation since *C. trachomatis* is associated with infertility and is a determining factor in decreasing the population density of indigenous communities. The situation is further aggravated by the detection of other STI markers, including reactivity to VDRL, persistence of HBV, lesions due to herpes simplex and the presence of *Neisseria* in children who had vaginal discharge [139].

The investigation of the prevalence of antibodies against *Chlamydia* was further pursued and included 430 HIV-1 carriers, of which 64.2% showed positive results. Of great relevance was the finding of 12.6% with IgM antibodies, demonstrating the presence of a recent or ongoing infection. A sample of positive persons showed seroreactivity by MIF only to *C. trachomatis* serotypes [54]. These prevalence rates are compatible with the wide spread presented by *C. trachomatis* among population groups seeking diagnosis of STIs [9,174]. The high prevalence of antibodies in a group who should be exerting basic STI preventive measures is a worrying matter, including the continuing risk behavior of sexual transmission of infectious agents. Although *C. trachomatis* infection is treatable, its clinical and laboratory diagnosis are not commonly performed, since most of the carriers of the infection are asymptomatic. Risk behaviors found were anal sex and unprotected sex with more than one partner per week. Such variables are also commonly associated with other STIs as well [35,155,175,176,177,178].

One of the epidemiologically interesting ecosystems in the Brazilian Amazon is the Marajó Archipelago, which has a low socioeconomic and education level and poor sanitary conditions that facilitate the spread of STIs. The difficulties in traveling within the archipelago and the distance from urban centers have resulted in social, political and economic isolation and, consequently, poor access to health, which contributes to increases in the disparities that affect the communities inhabiting the archipelago. A prevalence of antibodies against *C. trachomatis* of 30.9% was found in 1,217 people living in four municipalities located from north to south of the archipelago (Chaves, Anajás, Portel and São Sebastião da Boa Vista), with 6.7% showing evidence of recent or ongoing infection (presence of IgM antibodies) [157].

A sample of 393 women was investigated for the presence of cervical infection and 4.1% showed infections with genotypes D, E, F, Ia, J and B, commonly found genotypes in eye infections [13]. It should be noted that seroreactivity specific to serotypes B and Ba had already been detected in other locations in the Brazilian Amazon in the general population [179]. The percentage of people with active genital infection was also compatible with the frequency of those showing IgM antibodies against the bacterium [157].

Social (illiteracy and poverty) and behavioral (alcohol use, multiple sexual partners) variables continue to be important for the spread not only of *C. trachomatis*, but also of other STIs investigated [157]. The most relevant barrier for community development is the low educational level, and seroepidemiological investigations become essential to show the inability to control the spread of STIs [35,101,155]. Only a small number of persons have enough information to develop more complex activities, as well as read and understanding disease prevention measures. As a repetitive cycle, the Marajó Archipelago continues to be one of the most vulnerable regions in the state of Pará [180,181], where sometimes the family income does not exceed half the minimum wage [157].

Another important manifestation of *C. trachomatis* that cannot be omitted from this review is the presence of trachoma, a neglected disease that affects vulnerable populations and is a major cause of blindness by infectious agents [182]. Signs of active or inactive trachoma have been reported in 33% of the inhabitants of the Brazilian Amazon for more than 40 years [183]. Investigations by the VL showed that serotypes associated with trachoma were commonly found in several geographical areas [9,10,157,179] and serotype A, that used to have its geographical distribution limited to the Middle East and North Africa, was also detected in the Brazilian Amazon for the first time in a semi-isolated community [179]. This was probably introduced through contemporary human migrations [184].

From the serological point of view, it was possible to show in the Marajó Archipelago the extent of the spread of *C. trachomatis* and its serotypes [179]. Genotype B was also found among cervical specimens, which was also observed outside the country [185]. The finding of *C. trachomatis* serotypes/genotypes associated with trachoma came to confirm the need for disease surveillance in the archipelago [186,187]. In an initiative to eliminate trachoma, educational work was carried using the surgery, antibiotics, facial hygiene and education for better habits (SAFE) strategy after the diagnosis of cases. After eight years of follow-up, a marked reduction in the disease [187] and the vectors of transmission associated with poor hygiene was observed. The study proved that ocular transmission of *C. trachomatis* in areas with a low human development index needs public policies directed to the region and must include routine examination, immediate treatment, recognition of the associated risk factors and education campaigns in health with accessible language and adaptation to the local population, in addition to a continued investment in school education [157,187,188].

In the evolution of the investigation of *C. trachomatis*, as a relevant STI agent, and eye infections, experimental research focused on its role (along with *C. pneumoniae*) in the etiology of heart disease. The first approach was the demonstration of specific antibodies to *C. trachomatis* among patients with heart disease treated at a university hospital, with a frequency equal to or greater than that of the urban population in general [179]. Subsequent investigation showed the participation of *C. trachomatis* among patients with coronary artery disease with indication for coronary artery by-pass graft and patients with heart valve disease with indication for valve prosthesis implantation (mitral or aortic).

The multiplication of infectious agents may be the etiology of the chronic inflammation observed in the arterial wall of patients with atherosclerosis [189,190,191], and *C. pneumoniae* is one of the main agents hitherto demonstrated. *C. trachomatis* was detected in situ in 7.4% of the samples tested for the cryptic plasmid with four samples from the aorta and two from the mitral valve [14], and the in situ presence of *C. trachomatis* antigen by means of immunohistochemistry, in samples of aorta, valves and atheromatous plaque was demonstrated [12]. It should be stressed that genetic polymorphisms of proinflammatory cytokines and their participation in the microenvironment of the vascular and cardiac system have also been investigated and showed an intense local and systemic inflammatory reaction among the investigated patients [12,14,192]

## 9. Concluding Remarks

The Amazon region of Brazil is the largest area of the country, but the region is usually overlooked when control of sexually transmitted infectious agents is considered within health programs. The Virus Laboratory of the Federal University of Pará has maintained a continuous investigation of STIs since the second half of the 1980s, including descriptive and molecular epidemiological studies of HIV-1, HTLV-1/2, HBV, HPV, *T. pallidum* and *C. trachomatis*. The initial seroprevalence studies evolved for two or three decades, which helped to show the variations in the occurrence of the agents throughout the years and the urgent need to implement health services to most of the population groups. New outcomes of disease were investigated among urban, non-urban and more vulnerable population groups (FSWs, MSM, quilombolas and indigenous communities). The introduction of molecular biology techniques was useful to detect the occurrence and dissemination of genotypes and new strains and their association in the progression of disease with the numerous polymorphisms of the immunological and the inflammatory responses of the host.

Observing the enormous input of data, however, shows that there are still several gaps in the knowledge produced in the last 30 years and it is important to point out new directions for future investigations using sexually transmitted infectious agents as important models of human pathogens.

## Figures and Tables

**Table 1:** Prevalence rates and molecular epidemiology of HIV-1 and HTLV-1/2 in population groups of the Amazon region of Brazil.

Location	Year of Collection	Group	Prevalence Rate (%)	Subtypes	Reference
**HIV-1**					
Goiás	1980	MSM *, FSW ^§^	0.0	-	[28]
Pará	1974–1980	General population, blood donors, indigenous	0.0	-	[28]
Pará	1983	Gold miners	0.0	-	[29]
Pará	1996	Indigenous	0.6	B	[32]
Pará, Amapá	1997	Indigenous	0.1	B	[33]
Amazonas, Roraima	2009–2011	Indigenous	0.13	-	[34]
Pará	2012	General population	0.2	-	[35]
Pará	2005–2014	General population	0.48	-	[36]
Pará	2009–2010	Pregnant adolescents	0.3	-	[37]
Amazonas	2008	Pregnant women	0.6	-	[38]
Pará	2005–2006	FSWs	2.3	B, F1	[39]
Pará	2017	FSWs	15.3	-	[40]
Pará; Amapá	2013–2018	PWUD ^¶^	15.2	-	[41]
Piauí	2007	Elderly people	3.7	-	[42]
Pará	2009	PLHA	-	B, F1, C, D, CRF02_AG	[27]
Amapá	2009	PLHA	-	B, F1	[27]
Mato Grosso	2008–2009	PLHA	-	B, F1, C, D	[58]
Tocantins	2008–2009	PLHA	-	B, C, F1	[59]
Amazonas	2006–2007	PLHA	-	B, C, BF	[60]
Amazonas, Rondônia, Roraima	2011–2017	PLHA	-	B, C, F1, BF1	[61]
Pará	2007–2008	Pregnant women with HIV-1	-	B, F, C	[62]
Pará	2016	PLHA	-	B, F1, BF1	[63,65]
**HTLV-1/2**					
Pará, Amapá	1997	Indigenous	0.19–0.29	HTLV-2a	[33]
Pará	2009–2010	Pregnant adolescents	0.6	-	[37]
Amazonas	2008	Pregnant women	0.0	-	[38]
Pará	2005–2006	FSWs	1.7	HTLV-1a	[39]
Pará	1994–1996	PLHA ^¥^	4.0	HTLV-1; HTLV-2	[43]
Bahia	1997	PLHA	16.3	HTLV-1; HTLV-2	[44]
Pará	2005	PLHA	3.5	HTLV-1a; HTLV-2c	[45]
Pará	2016–2017	PLHA	1.4	HTLV-1a	[46]
Piauí	2007	PLHA	1.6	HTLV-1a; HTLV-2c	[47]
Pará	1983–1991	Indigenous	7.8	HTLV-2a	[79]
Pará	2000 (?)	Indigenous	-	HTLV-2c	[83]
Pará	2015	Indigenous	29.0	HTLV-2c	[82]
Amazonas	2015–2016	Patients with hematological diseases	0.32	HTLV-2c	[88]
Pará	2013-2018	PWUD	5.3	HTLV-1a, HTLV-2b, HTLV-2c	[98]
Pará	1999	Japanese immigrants	1,8	HTLV-1	[99]
Pará	2006	TSP/HAM	-	HTLV-1a	[100]
Pará	2002–2003	General population Marajó Island	0.11	HTLV-1a	[101]

MSM *: Men who have sex with men; FSW ^§^: Female sex workers; PWUD ^¶^: People who use illicit drugs; PLHA ^¥^: People with HIV/AIDS.

**Table 2:** Correlation of exposure and persistence levels of HBV among indigenous communities of the Amazon region of Brazil.

Exposure Level	Persistence Level	Indigenous Community	Exposure (%)	Persistence (%)
Low	Medium	Tiryó	6.4	3.2
		Asurini do Trocorá	5.1	3.1
		Kikretun	0	5.6
Medium	Low	Munduruku	22	0.6
		Yamamadi	17.9	0
Medium	High	Wayana-Apalai	26.3	14.2
		Yanomami	12.3	7.5
		Surui	24.2	11.3

**Table 3:** Correlation of exposure and persistence levels of *Chlamydia* among indigenous communities of the Amazon region of Brazil.

Exposure Level	Persistence Level	Indigenous Community	Exposure (%)	Persistence (%)
Low	Low	Mundurukú	20.4	3.3
Low	Medium	Arára Laranjal/Kurambê	27.7	7
Low	High	Tiriyó	11.5	33.3
Medium	Low	Kokrainimôro	55.9	1.9
Medium	Low	Asurini Kuatinemo	61	4
Medium	Medium	Cinta-Larga	47.1	6.2
High	Low	Awa-Guajá	90.7	2.6
High	Medium	Parakanã	81	5.9
High	Medium	Xicrin	81.5	9.5
High	High	Kubenkokrê	75.8	10.1
High	High	Yanomámi	87.6	21.1

## Data Availability

Not applicable.
